# Irrational herding persists in human-bot interactions

**DOI:** 10.1038/s41598-025-05534-8

**Published:** 2025-07-02

**Authors:** Luca Verginer, Giacomo Vaccario, Piero Ronzani

**Affiliations:** 1 Chair of Systems Design, ETH Zurich, Zurich, Switzerland; 2 Chair of Ecosystem Management, ETH Zurich, Zurich, Switzerland; 3 International Security and Development Center, Berlin, Germany

**Keywords:** Psychology, Human behaviour

## Abstract

We explore human herding in a strategic setting where humans interact with automated entities (bots) and study the shift in the behaviour and beliefs of humans when they are aware of interacting with bots. The strategic setting is an online minority game, where 1997 participants are rewarded for following the minority strategy. This setting permits distinguishing between irrational herding and rational self-interest—a fundamental challenge in understanding herding in strategic contexts. Moreover, participants were divided into two groups: one informed of playing against bots (informed condition) and the other unaware (not-informed condition). Our findings revealed that while informed participants adjusted their beliefs about bots’ behaviour, their actual decisions remained largely unaffected. In both conditions, 30% of participants followed the majority, contrary to theoretical expectations of no herding. This study underscores the persistence of herding behaviour in human decision-making, even when participants are aware of interacting with automated entities. The insights provide profound implications for understanding human behaviour on digital platforms where interactions with bots are common.

## Introduction

Herding, a phenomenon studied in ethology, psychology, and economics, refers to the convergent social behaviour where individuals align their actions without explicit coordination^1,2^. This behaviour involves making choices based primarily on popularity rather than expected utility. Examples of intentional exploitation of herding include claques (individuals hired to applaud or heckle at theatre performances) and shill bidders (individuals inflating auction prices by placing fake bids in cahoots with the seller).

However, this inclination towards collective alignment is not always rooted in manipulative intent. Herding can be rational when the group has better information than the individual^3^. Consider the simple act of choosing a restaurant in an unfamiliar city: the sight of a bustling establishment, with patrons waiting outside, can be a compelling indicator of the restaurant’s quality. Here, the individual assumes that the collective knowledge of the crowd surpasses their own limited information, leading them to align with the popular choice. Yet, this rational basis for herding becomes murkier in more complex scenarios, such as stock markets during economic bubbles. Investors, driven more by a fear of missing out and market sentiment^4,5^ than by objective analysis, invest in popular stocks^6^. This behaviour, although rooted in the perceived wisdom of the majority, can lead to inflated prices and result in devastating financial downturns when bubbles inevitably burst^7^.

Stock market bubbles are examples of *irrational* herding, a fascinating phenomenon caused by flawed human decision-making. This phenomenon manifests when the desire to fit in or avoid missing out overwhelms logical judgment. It can be attributed to psychological factors such as social proof and cognitive biases, which lead individuals to rely on readily available information, in this case, the collective actions of others, rather than conducting their comprehensive analysis. Therefore, there is a stark difference between rational and irrational herding: while the former is observationally equivalent to rational behaviour, the latter showcases how human heuristic guiding decision-making can be costly.

Both rational and irrational herding have a long history in behavioural research, reaching back to the Asch conformity experiments^8^. Depending on the alleged mechanism driving this collective phenomenon, it is known under different names, such as social proof^9^, conformity^8^, information cascades^10^ and social learning^11^. This conformity is considered positive as it reduces conflicts^12^ and increases accuracy^13^ but may^14^ or may not undermine the wisdom of crowds^15^. This observation raises societal concern to understand when herding is beneficial for the emergence of cooperation and its sustainability.

One of the inherent challenges in studying herding lies in the complex feedback loop between individuals and groups. An individual’s decisions influence the collective group behaviour, and in turn, this group behaviour impacts individual choices. This intricate interplay makes it challenging to discern the relative importance of individual versus collective influences.

This challenge in studying collective behaviour is not unique to humans. Ethologists encounter similar issues when studying animal behaviour and have developed methods to isolate the impact of the group on the individual. For example^16,17^, let animals, such as zebrafish and locusts, interact with computer-simulated conspecifics. By manipulating the behaviours of these virtual counterparts, researchers measure their impact on the live animals, shedding light on the link between individual and collective behaviour.

Building on this approach, our study uses scripted automated player—*bots* for short—to investigate how group behaviour influences individual choices and remove one side of the feedback loop: the individual on the group. To test for cooperative herding behaviour, we conduct a large-scale online experiment where participants played a minority game with bots. Moreover, by selectively informing half the participants about the automated nature of their opponents, we study the effect of perceived opponent nature (i.e., bot or human) on herding tendencies and the beliefs about their opponents. We refer to these two conditions as I (informed) and Not-I (not informed). Participants in the not informed condition were debriefed after the experiment was concluded.

Using bots to study prosocial behaviour is an “interesting yet underutilized resource”^18^. Indeed, humans attribute different degrees of anthropomorphism when interacting with robots^19,20^. Moreover, Sandoval et al.^21^ investigating strategic interactions of humans playing against bots in ultimatum and prisoner’s dilemma games, and find that cooperation with bots is lower, while reciprocity is unaffected. Also, Crandall et al.^22^ showed that in two-player repeated games (e.g., prisoner’s dilemma) bots interacting with humans achieve human-level cooperation. However, the effectiveness of using bots to increase cooperation is still debated^23^. This leads us to our first research question: Is there excessive cooperation when opponents are known to be bots?

**Fig. 1:**
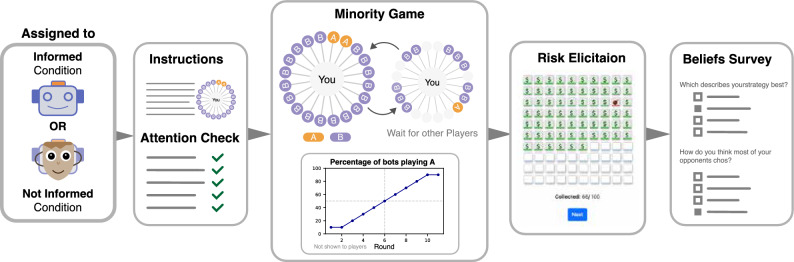
A flowchart of the five key stages of the experimental design. Panel 1: Random assignment of participants to either the informed condition (opponents referred to as “Bots”) or the not informed condition (opponents referred to as “Players”). Panel 2: Introduction and attention checks; Panel 3: a wheel of choices, denoting A for cooperation and B for defection, showing the choices of opponents (referred to as “Bots” in the informed condition, “Players” in the not informed condition), and the proportion of bot players choosing A; Note that we use the notation with A and B only in this panel containing snapshots of the game played by participants. For the remainder of the paper, option A, denoting cooperation, is reported with C, while B, denoting defection, is reported with D. Panel 4: The Bomb Risk Elicitation Task (BRET) to assess risk propensity; Panel 5: The survey on participants’ beliefs about decisions and opponents. Note: The only difference between the conditions is the replacement of the word “Bots” with “Players”.

In our experiment, participants engage in an iterated minority game, also known as anti-coordination game. In this game, participants must choose whether to cooperate (C) or defect (D), observing the popularity of each choice in the preceding round. In the minority game, alignment with the majority is not the rational strategy for maximizing payoffs. This property is pivotal: If participants follow the majority, it is indicative of herding rather than rational self-interest. Hence, it allows us to distinguish costly herding from rational choice. This leads us to the second research question: Is there irrational herding, and does it change when opponents are known to be bots?

From an experimental design perspective, we do not explicitly tell participants that they are playing a minority game. Instead, we present the game as a (weak) prisoner’s dilemma with a ‘bonus’ system. We introduced this so that the game’s minority rule would not be trivially apparent. The indirection encourages participants to focus on what others do. While this could introduce noise, it is part of the learning processes we are interested in, and crucially it does not alter the incentive structure and optimal strategies of the task. In a repeated (weak) prisoner’s dilemma, mutual cooperation is not a stable equilibrium as any player benefits from defecting unilaterally. Players are then informed about a bonus—awarded only to cooperators for each defector—which transforms the game into a minority game. The payoffs and bonus are calibrated so that when most players defect, the bonus awarded to each cooperator more than compensates for the lower base payoff, making cooperation the more profitable choice. Conversely, when the majority cooperates, the number of bonuses paid decreases, and defecting yields a higher payoff. In short, whichever strategy is chosen by the minority yields the greater return. This layer of complexity is intentional, challenging participants to discern the game’s mechanics and encouraging them to derive cues from the choices of others.

Each participant plays the iterated minority game against 20 other players, unaware of the pre-set total of 11 rounds, see Fig. 1. The other players are scripted bots whose programmed decisions are independent of participants’ choices. These bots gradually increase their cooperation, starting at 10% and culminating at 90% by the final round. Midway through round six, the bots’ strategies are evenly split. Up to this switching point, cooperation is the optimal strategy; afterwards, defection is. We chose a high number of opponents, as herding has been found to increase with group size^8,24,25^. We also use dynamic visualizations to make it seem like real-time competition, showing bot decisions after participants choose (see middle of Fig. 1 or the video of the game in the supplementary materials).

Post-gameplay, participants complete a risk elicitation task to control for potential confounding factors related to risk attitudes. We finally gather insights into their beliefs about their decisions and their opponents. A comprehensive breakdown of the experimental setting is detailed in the methods section.

Our study has two objectives. First, we probe how group dynamics, simulated through bots, influence individual choices. Second, we explore the nuances of human-bot interactions, investigating how awareness of competing against bots may change behaviour and beliefs.

Based on the above research questions, we pre-registered the following three hypotheses^26^ osf.io/njzas/:

### H1

*Excessive cooperation. Players will often play the sub-optimal strategy cooperate (C) even when the majority of the bots play C. We test this hypothesis separately in I and Not-I.*

### H2

*Herding. The proportion of players following the majority is significantly larger than 0. In other words, there are players playing D (and C) when the majority of bots play D (and C). We test this hypothesis separately in I and Not-I.*

### H3

*Human-bot interaction players in Not-I play more C than players in I.*

Assessing these hypotheses serves two purposes: it enriches our understanding of both human herding and human-bot interactions. Our results hold relevance for the architecture and governance of online platforms in a world becoming progressively digital and powered by artificial intelligence. For instance, bots are ubiquitous on platforms like X and Reddit, wielding significant influence over public dialogue and individual user conduct. Therefore, the implications of our study may inform discussions and policy-making aimed at ensuring ethical and transparent human-bot interactions on digital platforms.

## Results

### Excessive cooperation

**Fig. 2:**
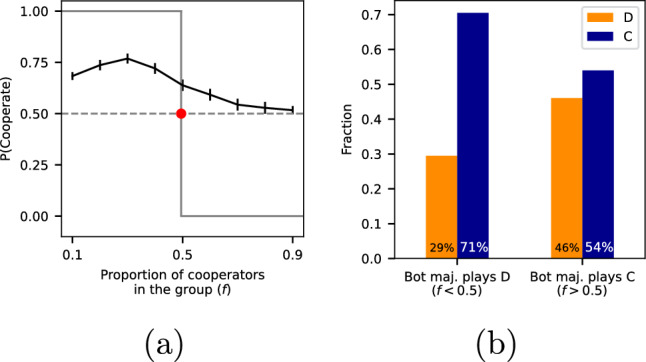
(**a**) The graph illustrates the likelihood of cooperation based on the percentage of bots that chose to cooperate in that round, represented by a black line. The error bars provide a 99% confidence interval. The grey line represents the ideal rational choice. The red point at P(C)=0.5 shows the anticipated response from both perfectly rational and boundedly rational agents when cooperation and defection yield identical payoffs (the switching point). The difference ($\Delta >0$) between the black line and the red dot indicates a prevalent trend towards over-cooperation. (**b**) The graph showcases the fraction of players choosing C or D in response to the majority bot behaviour. It reveals instances of irrational herding, where players make costly decisions to follow the majority bot choice, even when it was suboptimal in this minority game.

In the context of the minority game—where selecting the less popular option results in greater rewards—standard economic theory suggests that players choose the less common strategy for the highest expected return. Over time, perfectly rational players are expected to transition from cooperation (C) to defection (D) when cooperation becomes more widespread. We use *f* to represent the proportion of bots that choose to cooperate.

Figure 2 depicts the best response function for this game as a step function based on *f*: $P(C | f )=\Theta (0.5-f)$, i.e., players should choose C when $f<0.5$, and D otherwise. However, it has long been recognized, since^27^ groundbreaking work, that human decision-making is not perfectly rational. Bounded rationality offers a more flexible perspective, considering individual cognitive limitations and available information when making decisions.

Accordingly, in the bounded rationality framework, the probability of playing *C* transitions smoothly as a function of popularity rather than a step function. In a minority game, there exists a concept known as the ’switching point’. This is where options *C* and *D* are equally popular. This is illustrated by the red dot in Fig. 2. When the game reaches the switching point, we expect participants to choose *C* and *D* with equal probability.

If, instead, the probability of playing *C* exceeds $f=0.5$ when there is no majority, it points towards excessive cooperation. This would imply that, overall, participants favour cooperation more than would be expected in a perfect or bounded rational scenario. Moreover, when there is a large majority playing *C*, according to bounded rationality, we would expect *P*(*C*) to be significantly below 0.5. However, if it is significantly higher, participants may be inclined to follow the majority’s decision, leading to herding behaviour.

Our experiment provides evidence consistent with the predictions of herding. Confirming our first pre-registered hypothesis, we find that the average probability of cooperating at the switching point ($f=0.5$) is 63.9%, significantly higher than the expected 50% (*p*-value $< 0.001$, one-sample *t*-test). This implies a preference for *C* over *D*.

Also, in line with our predictions and confirming our second pre-registered hypothesis, a large fraction of the players follow the majority (herding) even if it is irrational (see Fig. 2b). Precisely, when the majority of bots chose D, players still chose D nearly one-third of the time. Likewise, cooperation persists even when it becomes the more costly choice. Despite rational or bounded rational expectations of no cooperation (0%), on average, 51.7% of participants still opted for cooperation, even when faced with a considerable majority of cooperators ($f=0.9$). With a 99.9% confidence level (*p*-value $< 0.001$, one-sample *t*-test), we can assert that the observed proportion is above 0%. This pattern suggests that, once established, excessive cooperation can be maintained over short periods.

### Risk aversion and decision-making

A plausible explanation for the excessive cooperation we observed could be risk aversion: participants, seeking to minimize potential losses or variability in outcomes, chose *C* as it offers lower variance in expected payoffs. If this were the case, the choice for *C* would not indicate cooperative intent but rather a strategy to mitigate risk.

However, our data counters this interpretation. When examining participants’ risk propensities (refer to the methods section for measurement details), we found no substantial correlation between risk aversion and the likelihood of choosing *C*. The correlation was slightly negative, as evidenced in Table S3 in SI. This suggests an intriguing conclusion: Participants with higher risk propensity were marginally more inclined to choose *C*.

In summary, while the choice of *C* can be seen as a risk-mitigating strategy, our findings indicate that risk aversion does not drive this behaviour. As observed in our experiment, excessive cooperation cannot be solely explained by participants’ risk propensities.

### Breakdown of strategy profiles

**Fig. 3:**
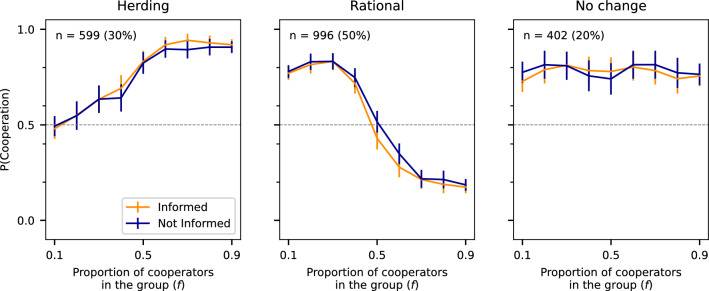
Illustrated here is the probability of cooperation, *P*(*C*), plotted as a function of the number of cooperating bots from the preceding round. The left panel depicts the average *P*(*C*) for scenarios where $P(C|f<0.5)<P(C|f>0.5)$, applicable to 30% of the participants. In the centre, the average *P*(*C*) is shown for cases where $P(C|f<0.5>P(C|f>0.5)$, representing 50% of the participants. On the right, the average *P*(*C*) is displayed for situations where $P(C|f<0.5)=P(C|f>0.5)$ corresponds to the remaining 20% of the participants. The two treatment groups, “Informed” (orange) and “Not Informed” (blue), show the difference in the probability of cooperating depending on whether they were uninformed about opponents being bots.

We propose ‘herding’ as a potential mechanism to explain the observed costly cooperation, i.e., cooperation even when the majority plays *C*. To test this, we look at the propensity to cooperate when the minority of other participants (bots) plays *C*, defined as $P(C|f<0.5)$, and the propensity to cooperate when the majority plays *C*, defined as $P(C|f>0.5)$. In other words, we examine the propensity to cooperate before and after the switching point ($f=0.5$), the moment when the majority of other participants (in this case, bots) switch from *C* to *D*. This enables us to deduce whether the participant follows the majority, indicating a “herding” behaviour, or the minority, suggesting a “rational” decision-making process.

To distinguish between herding and rational decision-making, we categorize participants into three types, based on their responses to previous decisions observed from their opponents. Formally,

- *Herding* Participants follow the *majority* more after the switching point, $P(C|f>0.5)< P(C|f<0.5)$
- *Rational* Participants follow the *minority* more after the switching point, $P(C|f>0.5)< P(C|f<0.5)$.
- *No change* Participant choose *C* irrespective of the proportion of cooperators, $P(C|f<0.5) = P(C|f>0.5)$.

Individuals assigned to the herding type consistently aligned their choices with the prevailing majority. In contrast, participants classified under the “no change” type do not respond to shifts in opponent behaviour. Finally, the rational type is characterized by participants who deliberately selected the minority option that yield the highest expected payoff.

Figure 3 illustrates the distribution of participant types. Of 1997 participants, 599 (or 30%) followed the majority, indicating herding behaviour. In contrast, 996 participants (or 50%) made what we classify as a “rational” choice, while the remaining 402 participants (or 20%) showed no discernible influence from the majority’s choice.

Excessive cooperation is most noticeable at the switching point ($f=0.5$), where herding participants cooperate with a probability of 82.8% and increase to 91.3% when the majority reaches $f=0.9$.

On the other hand, Fig. 3b illustrates that players identified as rational do not engage in excessive cooperation at the switching point. Rational players’ propensity to cooperate at the switching point is 47.5%, which is not significantly different from 50% (*p*-value=0.12, one-sample *t*-test). Therefore, we conclude that rational players are indifferent between the two options when no majority exists.

Turning to Fig. 3c, we see that participants who maintain a consistent cooperation probability throughout the game, no change, tend to cooperate more often. This observation corroborates prior findings on human pro-social behaviours. It also suggests that herding is not the sole factor contributing to excessive cooperation.

### Differences across informed and not-informed conditions

As part of our pre-registered analysis, we tested whether knowledge about the nature of opponents affects participants’ decisions. In one condition, we omitted the information that participants were playing against bots, while in the other, we explicitly informed participants about it (see Methods for details). Despite participants’ awareness of interacting with bots, their decisions appear to be unaffected.

This null result is illustrated in Fig. 3, where we plot the probability of choosing C as a function of cooperators in the group. In all three plots, the 99% confidence intervals overlap across conditions providing a negative answer to our third hypothesis. On average, the difference across conditions was around 1% (See Fig. S6 in SI). This null result is also suggested by a Bayesian test of association yielding Bayes’ factor below 1 (see paragraph “Testing for H3” and Table 1 in Methods for details).

It is worth noting that our experiment was sufficiently powered to detect a 10% effect size at a significance level of 0.01 ($\alpha$) and a power of 0.95 ($1-\beta$). This effect size, in our view and as stated in the preregistration, represents a meaningful level for detecting substantive behavioural differences between human-to-human and human-to-bot interactions.

Given this null result, we explore one other dimension that may differ between the conditions. Specifically, we consider response time as a metric of attention and effort. Examining response time—the time it takes participants to complete a round—we found no credible difference across conditions. This suggests that attention and effort remained consistent whether participants knew they were playing against bots or humans (see Fig. S5 in the SI).

This null result might raise the concern that participants ignored the information about the nature of their opponents. As shown in the following sections, we ruled out this possibility by showing significant manipulation effects on beliefs.

### Beliefs about own and opponents’ behaviour

**Fig. 4:**
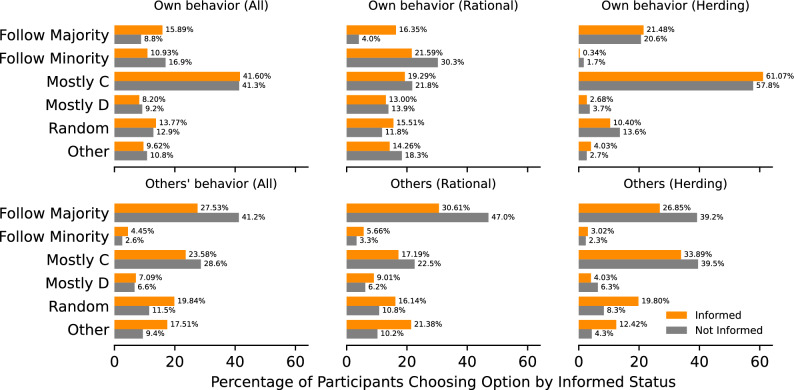
Results from participant surveys for all participants (left), those classified as rational (middle), and those classified as herding (right). The top row shows beliefs about their own behaviour, and the bottom row shows beliefs about their opponent’s behaviour. We separate the responses based on whether the participants were informed that they were playing against a bot or not.

We explore whether participants were aware of their own behaviour. In Fig. 4, we present responses to questions probing participants’ perceived strategy and their beliefs regarding opponents’ decisions by their informed status.

Responses from the post-experiment survey reveal that 41% of all participants believed they chose “Mostly *C*,” suggesting that their actual behaviour—cooperating more than expected–aligns with their perception of their own actions. This holds regardless of whether they believed they were playing against bots or not. Moreover, 27.53% of not-informed participants and 41.2% of informed participants believed that their opponents, who were bots, primarily “Follow(ed) the Majority,” while only 2.6% of not-informed participants and 4% of informed participants thought they were following the minority. This indicates that participants had a general understanding of their opponent’s strategies.

The second most common response was “Mostly C” (23.58–28.6%), although this is inaccurate as bots played *C* and *D* equally. However, “Mostly C” is a correct descriptor for the latter half of the game, suggesting that this belief may stem from a recency bias.

When analyzing “herding” participants—those who followed the majority—we find that 80% reported that they either “mostly played *C*” (59%) or “followed the majority” (21%) in their decision-making process. These reported beliefs align with their observed choices and a recency bias.

**Fig. 5:**
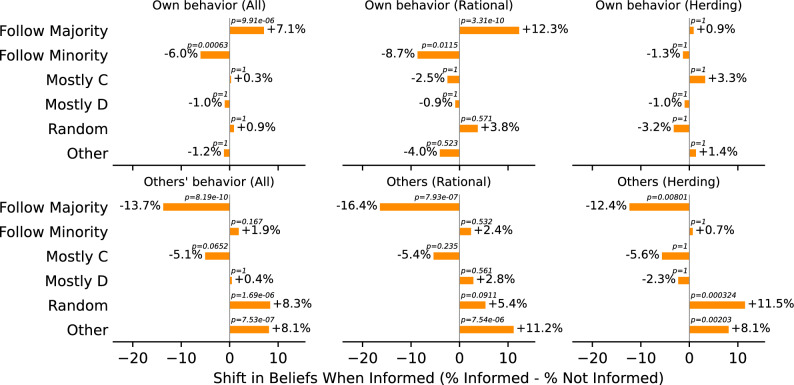
Multi-panel figure displaying the absolute changes in participants’ strategy choices when informed they’re playing against bots. Each bar represents the difference in percentage choosing each survey answer when informed versus not-informed. Positive (negative) values indicate an increase (decrease) in choice frequency of a specific answer. The top row examines beliefs about other’s actions, and the bottom row explores beliefs of participant’s own actions. Columns from left to right represent shifts for all, rational, and herding participants, respectively. *P*-values above bars are Fisher’s Exact Test results (two-sided), adjusted for multiple comparisons (Bonferroni). These are the differences of the percentages reported in Fig. 4.

An intriguing divergence in participants’ responses emerges when we consider whether participants were informed about their opponents being bots. In Fig. 5, we show the change in response to the final questions regarding the participants’ beliefs about their own and their opponents’ behaviour.

Participants’ beliefs about their opponents’ strategies did shift across conditions (bottom middle panel of Fig. 5). Specifically, informed participants moved away from believing that opponents primarily “followed the majority”, instead attributing more “random” or “other” strategies to them. This shift in beliefs is true across all strategy types (i.e., rational, herding and no-change).

The stark difference in beliefs can make us exclude the possibility that participants did not respond to the manipulation. Participants were randomly assigned to the Informed and Not-Informed conditions, so they were indistinguishable a priori. After the manipulation and playing the game, participants’ beliefs about their own strategies and, crucially, their opponents’ strategies are significantly different across conditions (see Fig. 5). These differences in beliefs indicate a quantifiable effect of the manipulation. Despite this, we find no significant change in decisions across conditions.

Rational players exhibited a significant shift in their beliefs about their own behaviour (top middle panel of Fig. 5). They believed they were more likely to follow the majority when informed about their opponents’ nature (i.e. when players knew that their opponents were bots). One plausible explanation for this observation is that informed, rational participants believed their opponents to be more sophisticated when they were bots. Hence, when participants were asked about their behaviour, they answered that they were following the majority of their sophisticated opponents. Since the actual behaviour is statistically indistinguishable across conditions (see middle panel of Fig. 3), we observe that rational participants were less coherent when informed. For the other participants (i.e., herding and no-change), no significant shift in the beliefs of their own behaviour is observed.

## Discussion

We investigated herding—the phenomenon of following the crowd—within the framework of strategic games. We designed an online experiment where participants played a minority game with automated players (bots), allowing us to test for cooperative and herding behaviours. Our experimental design has a twist: We informed half of the participants that their opponents were bots, while the other half remained uninformed. This manipulation allowed us to explore the impact of knowledge about their opponents’ nature on decision-making.

Contrary to theoretical predictions from game theory, the results reveal a robust preference for cooperation. Specifically, we observed that 64% cooperated even as 50% of their opponents cooperated, and 50% kept cooperating when 90% of their opponents cooperated, a costly decision in a minority game. In line with our pre-registered hypothesis, we found a high prevalence of herding behaviour (30% of the participants) despite a theoretical prediction of 0%. These results indicate a strong inclination towards conforming to the majority. Surprisingly, and against our expectations, this behaviour remained consistent even when participants were explicitly informed about their opponents’ non-human nature.

Despite their awareness of interacting with bots, we found no credible evidence to suggest that participants across conditions behave differently. Neither their cooperation nor herding behaviour were affected. This consistency underscores the robustness of these behavioural traits.

An intriguing aspect of our findings centres on the shift in beliefs. When participants knew they were playing against bots, they changed their beliefs about their opponents’ behaviour. Instead of thinking their opponents would follow the majority, participants assigned “random” or “sophisticated” strategies to the bots. Moreover, a specific type of participants (the more rational ones) also changed their beliefs about their own behaviour.

Despite these shifts in beliefs, there is little evidence that the participants’ decision-making was altered. Their tendencies to cooperate and follow the herd (majority) did not change. In other words, altering the opponents’ nature changes beliefs but not behaviour.

Herding can arise from numerous psychological and social motivations. Uncertainty about the optimal choice, cognitive constraints, and the cost of forming independent judgments can also compel individuals to follow others. While our experimental setup does not allow us to fully disentangle these forces, our findings reinforce that these motivations remain potent even in strategic interactions with non-human agents. Despite the many possible pathways leading to herding, it is clearly both prevalent and remarkably robust among our participants.

Our findings have potentially significant implications for designing and regulating online platforms in our increasingly digital and AI-driven era. For instance, bots commonly operate on social media platforms like X, influencing public discourse and user behaviour. Our study suggests that awareness of interacting with bots does not alter the ingrained human tendency to follow the majority. This suggests that bots can still manipulate decisions even if marked as such.

In an era of increasing digital sophistication, humans remain herd-prone—whether their peers are people or lines of code. This may have consequences in various domains. In financial markets, trading bots can intensify herd behaviour, prompting investors to follow prevailing trends despite rational analysis suggesting otherwise, thereby amplifying market volatility. Social media further exemplifies this phenomenon, as bots facilitate information cascades, fostering echo chambers that propagate misinformation and reinforce ideological polarization. Our results suggest that merely being aware of interacting with bots does not mitigate conformity. As AI systems evolve and bots become increasingly lifelike, this inclination risks to deepen—raising the prospect that humans will heed artificial voices, whether or not they recognize them as such. This has implications regarding bot disclosure policies, monitoring, and moderating interactions with bots to prevent potentially harmful herding behaviour.

Addressing these issues calls for effective mitigation strategies. One such approach is *transparent labelling*: clearly marking bot accounts and disclosing their affiliations (e.g., government, news agency) and crucially their intentions (such as influencing opinions or promoting products) could help reduce herding behaviour. Another safeguard involves the use of *bot activity alerts*, where platforms flag disproportionate bot activity on specific topics to help users recognize when the apparent majority may be artificially inflated. However, recent developments on platforms like X and Facebook suggest a retreat from such moderation policies.

Moreover, to understand the effectiveness of interventions, further research is necessary. Future studies should systematically test combinations of transparency cues (i.e., labelling), intent disclosures, and behavioural nudges. Understanding the effectiveness and unintended consequences of such interventions is critical to designing effective solutions to counter harmful herding (e.g., manufactured consensus) and safeguard independent judgment.

While the experiment provides valuable insights, it is also crucial to consider its limitations. Though carefully designed, the experimental setting is a simplified model of real-world social interactions. Online experiments, such as ours, offer anonymity that influences the applicability of the findings to real-world scenarios where social norms play a more substantial role.

Moreover, comprehension difficulty or (mis)understanding of the rules of the game might influence the observed behaviours. To not have a trivial minority game, we introduced the bonus system and omitted to describe the strategy of the bots. This introduced uncertainty and made it challenging fr participants to determine the best response. As a result, it may have affected their comprehension and influenced their observed behaviours. To address this, we used attention checks to ensure that participants understood the basic mechanism of the game before proceeding. However, the complexity of the setup and potential misunderstandings may still have influenced their behaviour. Future research should, for example, use the number of failed attention checks as a proxy for misunderstanding and explore possible links with herding behaviour.

Furthermore, our research primarily focused on how group behaviour influences individual decision-making without considering how individual decisions might, in turn, affect the group’s behaviour. Understanding this interplay is an exciting avenue for future research.

In conclusion, our study offers a novel perspective on the robustness of human cooperative and herding tendencies within the context of strategic games, even when the nature of the opponents is known to be artificial. These findings contribute to a deeper understanding of the complex mechanisms driving human decision-making. They also underscore the need to further explore this phenomenon, particularly in online environments, where humans and bots interact regularly. Ultimately, the insights gained from this research could prove instrumental in informing the design and regulation of our increasingly AI-driven digital landscapes.

## Methods

### Participants

All studies were approved by the ETH Zurich Ethics Commission (approval number 2022-N-37). All methods were performed in accordance with the relevant guidelines and regulations. All participants were at least 18 years old and gave their informed consent beforehand.

Participants were recruited through Prolific and paid an average rate of £9.57 per hour. Participants’ final earnings depended upon their choices within the experiment. Among the rounds played, one round was selected at random to determine their payoff for the task.

All participants were residents of the UK at the time of data collection, and had indicated in their Prolific profile that they were willing to participate in experiments involving potential deception (87% of participants on Prolific agreed to this). This must always be disclosed during the debriefing, as was done in this study. Prolific collected this information as part of user sign-up, not in proximity to our experiment (in many cases, several months prior); therefore, it is unlikely that the deception aspect was salient for participants during the experiment.

The sample provided by the recruitment platform was balanced on sex. Our target sample size was 2000 participants, guaranteeing a power of 95% or higher to test the pre-registered hypotheses; see the pre-registration on OSF^26^ and section “Sample Size and Power Analysis” below. After exclusion criteria and removing dropouts, the resulting sample is 1997.

### Experiment setup

Our experiment adopts a 2 by 1 design, reflecting two conditions wherein the manipulated factor is the participants’ awareness of their interactions with bots. Participants were randomly assigned to:

- *Informed condition* Participants are explicitly informed of their engagement with bots.
- *Not informed condition* The interaction with bots is concealed, referring to them simply as “players.”

Note, the only practical difference across the two treatment conditions was the change of the word “Bot” to “Player”, no other aspect of the game was altered. Videos and screenshots of the game, as shown to participants, are available in the Supplementary Materials.

2256 participants were recruited via Prolific. Prolific sent out more than 2000 invitations, and more than 2000 responded in sequence. Some clicked on the invitation but did not start the study (they never saw our experiment) due to a time-out, which reset the invitation counter and allowed others to join later. In total, this happened 256 times. Prolific delivered exactly 2000 participants who started the experiment as requested; however, 3 of those who started the online experiment dropped out (1 did not continue past the instructions, and 2 dropped out during the first two rounds).

1128 were assigned to the “Not Informed” condition and 1128 to the “Informed“ condition. 988 and 1009 participants assigned to the “Informed” and “Not Informed” condition respectively finished the experiment.

*Instructions and comprehension checks* During the initial phase of the experiment, all participants are presented with instructions and asked to complete comprehension questions regarding the task’s rules and the calculation of payoffs. In the event of errors in the comprehension checks, participants are asked to check instructions and provide revised answers. They can advance to the next phase only when all the answers are correct.

*Minority game* After completing the comprehension checks, participants are told they are placed in an ongoing game where they know they will play multiple rounds. However, they are unaware of the exact number of rounds (set to 11). In each round, players observe the strategy played by other participants in the previous round, along with the payoff matrix. Participants choose between option C and option D for each round, corresponding to “collaborate” or “defect”. In the game we decided to use the labels A and B instead of C and D to avoid attributing any positive or negative connotations to the available options, where C “collaborate” may be perceived as a virtuous choice by some participants. In other words, the readers of this manuscript will find the labels C and D, whereas the game participants have found the labels A and B.

Participants played this game simultaneously against 20 other bot players, and the payoffs were determined according to the following matrix, as presented to the participants:

$$\begin{aligned} \begin{array}{lccc} & \textrm{C} & \textrm{D} & \textrm{Bonus} \\ \textrm{C} & 6 & 0 & +4 \\ \textrm{D} & 10 & 0 & +0 \\ \end{array} \end{aligned}$$

In this matrix, the rows represent the player’s strategy, and the columns represent the strategies of the 20 bot players. The ’Bonus’ column indicates an additional payoff for a player choosing C, for every bot choosing D.

From a game theoretical perspective, this matrix is equivalent to the following simplified form:

$$\begin{aligned} \begin{array}{lcc} & \textrm{C} & \textrm{D} \\ \textrm{C} & 6 & 4 \\ \textrm{D} & 10 & 0 \\ \end{array} \end{aligned}$$

The entries in the matrix represent the payoffs to the player for each combination of strategies. The representation shown to participants, including the ’Bonus’ column, was designed to increase the effort required to understand the payoffs, thereby encouraging participants to seek cues from the decisions of others. In each round, players observed the strategies played by other participants in the previous round, along with the payoff matrix, and chose between choices C and D.

It is well established that in repeated games, individuals condition their strategies on observed play from previous rounds [camerer2011behavioural,^28–31,32^]. For instance, by observing five cooperators in one round, participants naturally anticipate a similar number of cooperation in the next round, especially when there is no evidence of abrupt environmental changes. Also, in line with learning models^29,31^, participants perceive and exploit such stable trends–here, a consistently high level of cooperation–rather than assuming the scenario might randomly change from one round to the next. The experimental design used in this paper ensures that the proportion of bots playing C changes smoothly (by exactly one in each round). This scripted, smooth, incremental change creates a predictable progression, leaving no indication of abrupt or random shifts in opponent behaviour.

*Testing for H1* In the first pre-registered hypothesis, we aim to test whether excessive cooperation exists. To do this, we check the average probability of cooperating at the switching point ($f=0.5$). We find that this probability is 63.9%, which is higher than the expected 50%. To test whether this difference is significant, we use a t-test. The 99% confidence interval (indicated via error bars) does not include 50%, confirming the significance of the difference (see Fig. 2).

*Testing for H2* The second pre-registered hypothesis is about irrational herding, i.e., about the proportion of participants following the majority even if it is costly. To check for this, we perform the following analysis. For each participant *i*, we compute two quantities: (1) the fraction of times the participant chooses *C* when the majority of bots played *D* ($P_i(C | f < 0.5)$), and (2) the fraction of times the participant chose *C* when the majority of bots played *C* ($P_i(C | f > 0.5)$). Then, we verify that the fraction of participants following the majority ($|\{i: P_i(C | f< 0.5) < P_i(C | f > 0.5)\}|/N$ where *N* is the sample size) is different from 0. This fraction is our estimate for the tendency to herd in the population. We find that this fraction is around 30%, implying that about 600 participants out of 2000 followed the majority even if it is costly.

*Testing for H3* This section presents empirical tests for our third hypothesis (H3) and its sub-hypotheses (as listed in the pre-registration). Specifically, we test the following sub-hypotheses:

#### H3.1

*Players who are not informed about the nature of their opponents (Not-I) cooperate more than informed players (I) (*$\beta _2 < 0$ *).*

#### H3.2

*The effect observed in H3.1 intensifies as the proportion of bots playing C increases. In other words, players in I will cooperate less when the fraction of bots playing C increases (* $\beta _3 < 0$ *) compared to players in Not-I.*

#### H3.3

*The proportion of herding players in Not-I is higher than in I.*

To conduct these tests, we employ both regression analysis and a $\chi ^2$ test in accordance with our pre-registration.

The dependent variable in the regression analysis is the participants’ decision to cooperate or not, represented as 1 or 0. The independent variables include the proportion of bots cooperating (referred to as *f* in the main text), ranging from $\frac{2}{20}$ to $\frac{18}{20}$, and whether the participants were ’Informed’ or ’Not informed’ about their opponents nature. Formally, the regression model is expressed as:

$$\begin{aligned} \text {Cooperate} =&\,\beta _0 + \beta _1 \times \text {Prop. Cooperators}+ \beta _2 \times \text {Not Informed} + \\&+\, \beta _3 \times ( \text {Prop. Cooperators} \times \text {Not Informed}) + \text {controls} \end{aligned}$$

If $\beta _2 < 0$, we find support for H3.1, suggesting that players in Not-I are more inclined to cooperate than those in I. If $\beta _3 < 0$, H3.2 is supported, indicating that players in I are even less likely to cooperate when more bots cooperate.

**Table 1:** Estimation results for factors influencing cooperative behaviour.

	Random effects	Pooled
(Intercept)	$$0.615^{***}$$	$$0.615^{***}$$
	(0.029)	(0.019)
Prop. Coop.	$$-0.256^{***}$$	$$-0.256^{***}$$
	(0.015)	(0.016)
Not informed	0.022	0.022
	(0.014)	(0.013)
Prop. Coop. and not informed	-0.040	-0.040
	(0.020)	(0.022)
% of Boxes collected	0.032	$0.032^{*}$
	(0.023)	(0.014)
Prior approvals	$$0.021^{***}$$	$$0.021^{***}$$
	(0.004)	(0.003)
Negotiation experience: Yes	0.009	0.009
	(0.011)	(0.007)
$\hbox {R}^2$	0.033	0.031
Adj. $\hbox {R}^2$	0.033	0.030
Num. obs.	21,967	21,967

The table presents logistic regression estimates from two models to identify determinants of the outcome variable ‘Cooperate.’ Both models include an interaction term between ‘Prop. Cooperators’ and ‘Not Informed’ while controlling for ‘Prior Approvals,’ ‘Negotiation Experience,’ and ‘Boxes collected’. The ‘Random Effects Model’ utilizes a random-effects logistic regression approach, and the ‘Pooled Model’ is based on a pooled logistic regression framework. The analyses are conducted on panel data indexed by ‘ParticipantID’ and ‘Round.’ See appendix for covariate definition.

$$^{***}p<0.001$$; $$^{**}p<0.01$$; $$^{*}p<0.05$$

Table 1 reports the results of the regression analysis. Neither $\beta _2$ nor $\beta _3$ are statistically different from zero. As such, we fail to reject the null hypotheses for H3.1 and H3.2, concluding that there is no significant difference in cooperative behaviours across the conditions.

To investigate H3.3, as stated in our pre-registration, we apply a $\chi ^2$ test to compare the proportions of cooperators and herding players across conditions and fail to reject the null hypothesis for H3.3, see Fig. S6 in SI.

For a detailed explanation of the $\chi ^2$ test and Bayes factor t-test methods employed, please refer to the appendix section titled “Chi-Square and Bayes Factor Analysis for Comparing Cooperation Across Conditions”.

*Sample size and power analysis* We aimed for a total sample size of 2000 participants, with an equal distribution between control and treatment groups. This size was chosen to reliably detect a minimum effect size of 10% with a significance level ($\alpha$) of 0.01 and a statistical power of 0.95, thereby minimizing the risks of Type I and II errors.

The target effect size was based on the scenario where the probability of choosing *C* (*P*(*C*)) in the control group is 0.5, as this represents the most challenging condition to detect an effect. To validate our choice, we employed Stata‘s power twoproportions command. The power analysis confirmed that a sample size of 2000 participants provides sufficient power to detect an effect size of at least 9.5% under the specified conditions, aligning with our pre-registration commitments (see Fig. S7 in SI). Note that when the *P*(*C*) of the control group deviates from 0.5, the sensitivity for detecting smaller effect sizes increases.

*Risk attitude* To assess risk attitude, we employed the bomb risk elicitation task (BRET)^33^. In this task, participants decide how many boxes to collect out of 100, with one of the boxes containing a bomb. Earnings increase proportionally with the number of boxes accumulated, but if the box with the bomb is also collected, earnings become zero. The BRET requires minimal numeracy skills, avoids data truncation, and enables precise estimation of risk aversion and risk-seeking behaviour^33^. We used the number of boxes collected in BRET to estimate a constant relative risk aversion (CRRA) parameter that quantifies the individual’s level of risk aversion and the results are available in the Appendix A.5. The key statistics of the boxes collected are: mean 49.6, std 22.7, min 4 and max 100.

*Beliefs and manipulation check* Once the minority game and the BRET are completed, participants are asked to express their beliefs regarding the strategy they employed during the minority game and the strategy used by the bots (or other players in the not-informed condition). Participants choose between the following options, answering the question: “Which describes your strategy best?”. With the following options: “I mostly played C (i.e., bonus choice)”, “I mostly played D (i.e., no bonus choice)”, “I followed the minority”, “I followed the majority”, “I randomly choose between C and D” or “I followed a different strategy”. Note that the labels presented to the participants were not C and D, but A and B.

Moreover, they are asked about their beliefs about their opponents: “How do you think most of your opponents chose?” with the analogous answers: “They mostly played C”, “They mostly played D”, “They followed the minority”, “They followed the majority”, “They randomly choose between C and D” or “They followed a different strategy”. Note that the labels presented to the participants were not C and D, but A and B.

We decided not to include a manipulation check (e.g., asking about the nature of the opponents before the game) because this would have primed the participants–especially those in the non-informed condition–to focus on their perception that their opponents were not human or were somehow “different.” Therefore, we could only conduct it at the end, and asking about differences in perceived opponent strategies was a neutral way to assess whether their perception was indeed affected.

*Demographic characteristics* Table 2 provides an overview of the demographic characteristics of the 1997 participants in the study. The data includes the total number of approvals and rejections, the approval rate, the age distribution, and the sex balance (coded as 1 for male). The participants’ approval rate reflects how often their submissions were included in experiments, as they provided quality answers; this rate is high at 99.43%. The age of the participants ranges from 18 to 84, with a mean age of 39.13, and the sample is balanced with respect to sex.

**Table 2:** Descriptive statistics of 1997 participants.

	Mean	Std	Min	25%	50%	75%	Max
Approvals	590.67	482.68	1	182	499	874	3072
Rejections	3.46	4.16	0	1	2	5	48
Approval %	99.43	1.02	94	99	100	100	100
Age	39.13	13.13	18	29	37	48	84
Male	0.50	0.50	0	0	1	1	1

## Supplementary Information

Supplementary Information.

## Data Availability

The online task was built in oTree^34^ and the code is available from GitHub (https://github.com/sg-dev/herding-game). Analyses were conducted in R (https://www.r-project.org/), Stata (https://www.stata.com/), and in Pandas (https://pandas.pydata.org/). For the Bayesian analysis, we used the BayesFactor R-package. The code for the analysis is available from GitHub (https://github.com/sg-dev/herding-analysis). The raw data collected during the experiment is available from the Open Science Foundation (OSF) repository of this project at https://osf.io/njzas/.
