# Supplementary material for: Irrational herding persists in human-bot interactions
Source: Sci Rep. 2025 Jul 2;15:22828. doi: 10.1038/s41598-025-05534-8 (PMC12214505; doi:10.1038/s41598-025-05534-8)
Supplement: Supplementary file 1 — Supplementary Information. [file 41598_2025_5534_MOESM1_ESM.pdf]

Supporting Information for

The Robotic Herd: Using Human-Bot Interactions to Explore Irrational Herding

Corresponding Author

This PDF file includes:

Supporting text
Figs. S1 to S8
Tables S1 to S4

Supporting Information Text

Strict Strategy Definitions. To further explore participants' choices and highlight their heterogeneity, we introduce "strict" and "relative" variants of the previous classes.

- **Strict Herding**: Participants consistently follow the majority, indicated by $P(C|f < 0.5) < 0.5$ and $P(C|f > 0.5) > 0.5$
- **Relative Herding**: Participants who follow more the majority after the switching point, but do not meet the criteria for 'Strict Herding',
- **Strict Rational**: Participants who consistently follow the minority, indicated by $P(C|f < 0.5) > 0.5$ and $P(C|f > 0.5) < 0.5$
- **Relative Rational**: Participants follow more the minority after the switching point, but do not meet the criteria for 'Strict Rational'.

Fig. S1 discusses the relative proportion of these four variants. Out of 1,997 participants, 180 (or 9%) can be classified as strict herding participants and demonstrate an average cooperation probability of 30% prior to the switching point. Cooperation increases to 86% when the vast majority cooperates (see top-left panel in Fig. S1).

Looking at strict rational participants, we see a textbook example of bounded rationality (see top-right panel in Fig. S1). Initially, participants cooperate with a probability of approximately 80%. However, this probability sharply declines to less than 20% when the majority of bots begins to cooperate.

We observe that relative rational participants after the switching point still cooperate a lot (see bottom-right panel in Fig. S1). This means that they also contribute to excessive cooperation. Moreover, we observe that relative herding participants cooperate a lot before the switching point (see bottom-left panel in Fig. S1). This suggest that they start out with a high propensity to cooperate and follow the cooperation trend of the bots.

Beliefs about Own and Other's Behaviour. Understanding participants' self-perception and their views on their opponents' strategies is critical for interpreting the results of the minority game. In this section, we present a detailed breakdown of participants' answers to questions regarding their own and their opponents' perceived behavior.

Table S1 illustrates how participants perceived their own choices. From the table, it can be seen that participants often categorized their strategies in various ways, such as following the minority, the majority, or even playing randomly.

Table S2 provides an overview of how participants perceived the strategies of their opponents. This understanding is critical as it can give insights into how players anticipated and reacted to other's actions in the game.

Impact of Information on Participants' Perceptions of Behavior. Figure S3 provides a comparative view of participant beliefs about personal and others' behaviors under two distinct conditions: informed of playing against bots (gray bars) and not-informed (dark orange bars). Various strategic categories are identified on the y-axis, and the x-axis shows respective percentages.

The top row, about own behavior, reflects consistent personal behavior beliefs across conditions. Participants seem to maintain a stable understanding of their own strategies, regardless of whether they know they are playing against bots.

The bottom row reveals significant perceptual variations. When not-informed, participants more often thought their opponents were following the majority. Conversely, when informed that their opponents were bots, they attributed more "random" or "other" strategies to them.

These findings highlight the nuanced ways in which information about the nature of opponents can shape player perceptions and beliefs. While personal strategies remain consistent, the understanding of others' strategies shifts significantly, demonstrating the power of context and information in shaping strategic decision-making.

P-values displayed in Fig. **??** in the main text shown above the bars are derived from Fisher's exact test (two-sided). They indicate the statistical significance between conditions for each strategy. To account for the multiple comparisons, we apply the Bonferroni correction to control the Type I error rate.

Time to Answer. The analysis of the time to answer provides insights into participants' decision-making processes during the game. It captures the time taken by each participant to make a decision, starting from the moment they see the final decisions and payoff from the last round. By examining the decision-making time across various rounds, we can gauge how participants' learning and effort evolved throughout the experiment.

The Fig. S4 represents the average decision-making time per participant for each round, providing a snapshot of the time needed to understand, evaluate, and act upon the information received. This data helps identify any potential learning effects or fatigue that might influence decision-making as the game progresses. However, given the overlapping 99% standard error, we can conclude that there is no credible evidence for differences across types.

The comparative analysis between the informed and not-informed conditions could help to identify systematic difference in effort or learning. Despite differences in awareness regarding the engagement with bots, we found little evidence of differences in the time taken to answer between the two groups, see Fig. S5

The lack of significant difference in response times between the two conditions suggests that knowledge of playing against bots may not substantially influence the immediacy or deliberation in decision-making. This aligns with the broader findings of the study, where awareness of the bot players did not dramatically alter the actual decisions made by the participants.

	No change	Herding	Rational	Rel. H	Rel. R	All
Mostly D	5.22	5.00	12.98	2.39	15.24	8.71
Other	5.22	8.33	19.47	1.19	4.76	10.22
Follow Majority	5.22	38.33	11.32	13.60	4.76	12.32
Random	14.68	22.78	12.47	7.40	17.62	13.32
Follow Minority	3.23	2.22	30.53	0.48	9.52	13.97
Mostly C	66.42	23.33	13.23	74.94	48.10	41.46
Total	100.00	100.00	100.00	100.00	100.00	100.00

Table S1. Proportion of answers about own choices.

	No change	Herding	Rational	Rel. H	Rel. R	All
Follow Minority	2.49	2.78	4.83	2.63	2.86	3.51
Mostly D	7.71	5.00	6.23	5.25	12.38	6.86
Other	15.67	8.33	16.54	8.35	11.90	13.42
Random	23.63	15.56	11.58	13.37	20.00	15.62
Mostly C	25.62	27.22	18.07	40.81	27.14	26.14
Follow Majority	24.88	41.11	42.75	29.59	25.71	34.45
Total	100.00	100.00	100.00	100.00	100.00	100.00

Table S2. Proportion of answers about other choices.

Probability to Choose Cooperation and Risk Attitude. We employed a linear probability regression model to investigate the factors influencing the probability of choosing Cooperation (C) among participants. Two separate models (Model 1 and Model 2) were constructed to examine different sets of explanatory variables.

Model Variables:

- **CRRA:** The Constant Relative Risk Aversion parameter obtained from the BRET task, included in Model 1 but not Model 2. It quantifies the participant's level of risk aversion.
- **Boxes Collected:** This variable, used in Model 2, represents the number of boxes collected by a participant in the BRET.
- **Male:** A binary variable indicating the gender of the participant.
- **Log(Age):** The natural logarithm of the participant's age.
- **Education:** Categorical variables representing different education levels of the participants.
- **Negotiation Experience:** A binary variable representing whether the participant has negotiation experience or not.
- **Charitable Giving:** Categorical variables capturing different ranges of charitable giving in British Pounds.

Model Summary: Model 1 includes CRRA as an explanatory variable, while Model 2 replaces it with Boxes Collected. Both models consider demographic and personal attributes like gender, age, education, negotiation experience, and charitable giving.

The R-squared values indicate the proportion of the variance in the dependent variable that is predictable from the independent variables, while the adjusted R-squared accounts for the number of predictors in the model. The number of observations for both models is 1997.

These models provide insights into the relationships between individual characteristics and the propensity to choose Cooperation (C) in the strategic game, offering a better understanding of how different factors may contribute to this choice.

Effect across behavioural types When comparing the average risk propensities of participants across different behavioural types (e.g., herding), we found no credible evidence of differences, as shown in Table S4.

Chi-Square and Bayes Factor Analysis for Comparing Cooperation Across Conditions. As pre-registered, we employed χ^2 tests to assess whether the proportions of participants choosing to cooperate ($P(C)$) differed between the 'Informed' and 'Not Informed' conditions.

To complement our frequentist approach, we ran a Bayesian test of association using the R package `BayesFactor`.

From this test, we obtain a Bayes Factor (BF), which serves as a Bayesian counterpart to the p-value, allowing us to quantify the strength of evidence for the null hypothesis—that there is no difference in $P(C)$ across conditions.

Specifically, we obtain the contingency table for each cooperation level (f) for the two decisions (C and D) across the two conditions. For each contingency table (one per cooperation level), we compute first the p-value of the χ^2 using R and then the BF using the `contingencyTableBF` function from the *BayesFactor* package. Specifically, we assume default priors using a joint multinomial sampling plan (`indepMulti"`).

We show the results in Figure S6. This figure plots the difference in $P(C)$ at different levels of bot cooperation. Below each point, we annotate both the corresponding p-value (χ^2 test) and BF. Importantly, our analyses did not find evidence for an effect on $P(C)$ across conditions. All p-values exceeded 0.19, and all Bayes Factors are substantially below 1.

Aggregate Analysis We extended our analysis to explore participants' behavior both before and after the 'switching point' ($f = 0.5$), which is the moment when 'Cooperate' ceases to be the optimal strategy. Consistent with our earlier findings, the data shows no significant effect on cooperation rates. Specifically, we observed only a negligible 1% difference in the rate of cooperation after the switching point (p-value of 0.16 and Bayes Factor of 0.06). Similarly, participants' behavior prior to the switching point also showed an inconsequential difference in cooperation rates—less than 1%—with a p-value of 0.36 and a Bayes Factor of 0.04.

Power Analysis.

	Probability to chose C	
	Model 1	Model 2
(Intercept)	0.365^{***}	0.398^{***}
	(0.069)	(0.067)
CRRA	0.013	
	(0.008)	
Coxes Collected		0.000
		(0.000)
Male	-0.027^{*}	-0.026^{*}
	(0.010)	(0.010)
Log(Age)	0.048^{**}	0.050^{**}
	(0.016)	(0.016)
Education		
Don't know / not applicable	-0.153	-0.151
Graduate degree (MA/MSc/MPhil/other)	0.018	0.017
High school diploma/A-levels	0.018	0.018
No formal qualifications	0.102	0.104
Secondary education (e.g. GED/GCSE)	0.025	0.025
Technical/community college	0.042	0.042
Undergraduate degree (BA/BSc/other)	0.039	0.038
Negotiation Experience		
No	0.009	0.009
Yes	0.006	0.005
Charitable Giving		
£1-£50	0.023	0.023
£50-£75	0.006	0.006
£75-£100	0.077^{***}	0.077^{***}
	(0.022)	(0.022)
£101-£200	0.021	0.022
£201-£500	0.008	0.009
£501+	0.033	0.034
R^2	0.024	0.023
Adj. R^2	0.015	0.015
Num. obs.	1997	1997

$^{***}p < 0.001$; $^{**}p < 0.01$; $^{*}p < 0.05$

Table S3. Linear Probability Regression.

	$\Pr(C)$	(SE)
(Intercept)	3.841	$(0.037)^{***}$
Herding	0.102	(0.057)
Pure rational	-0.027	(0.039)
Relative herding	-0.015	(0.044)
Relative rational	0.018	(0.054)
No Change	-0.005	(0.028)
Male	0.046	(0.028)
R^2	0.004	
Adj. R^2	0.001	
Num. obs.	1997	

$^{***}p < 0.001$; $^{**}p < 0.01$; $^{*}p < 0.05$

Table S4. Linear Probability regression on the proportion/propbability of participant to choose C.

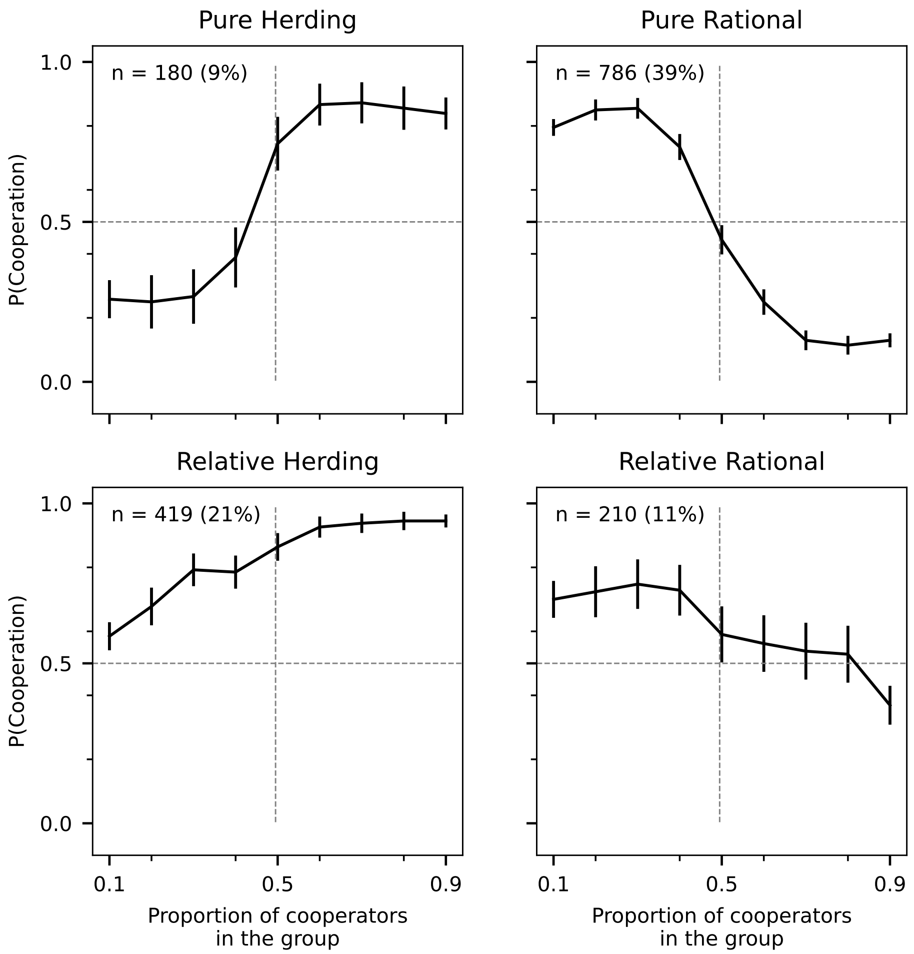

Fig. S1. The probability of cooperating as a function of the number of cooperating bots in the previous round. On the top-left, we consider the average $P(C)$ for the strict herders. On the bottom-left, we consider the average $P(C)$ for the remaining herders. On the top-right, we consider the average $P(C)$ for the participants following the bounded rational strategy. On the bottom-right, we consider the average $P(C)$ for the remaining participants following the minority.

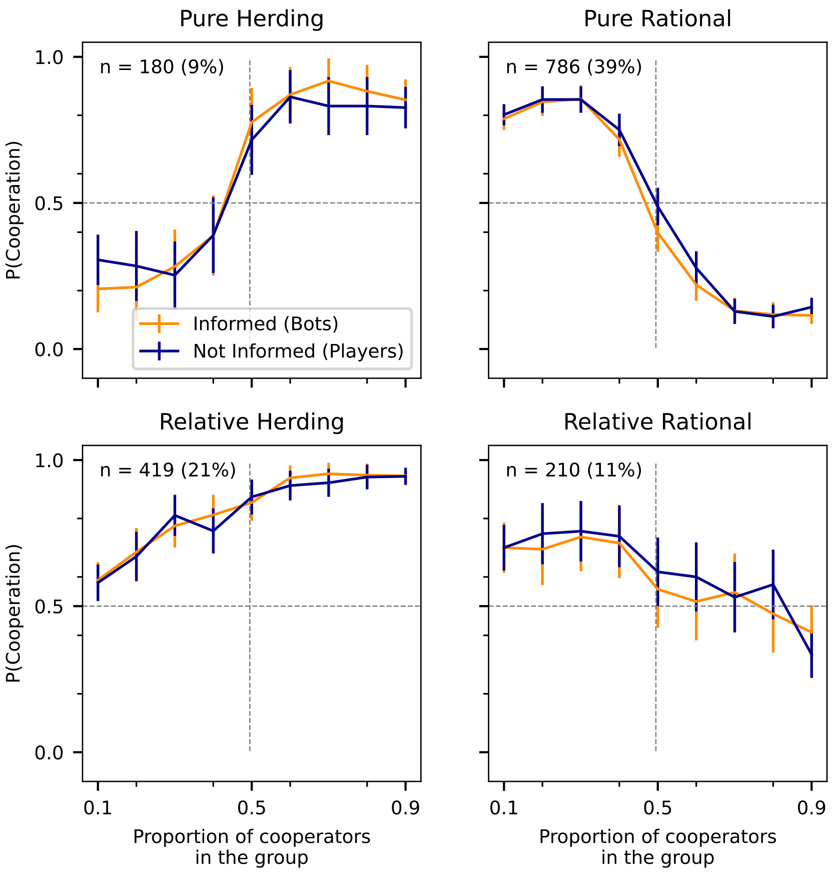

Fig. S2. Probability to play C by condition the participants were assigned to: they were informed that their opponents are bots, and they were not informed during the game (they receive this information only in the debriefing stage).

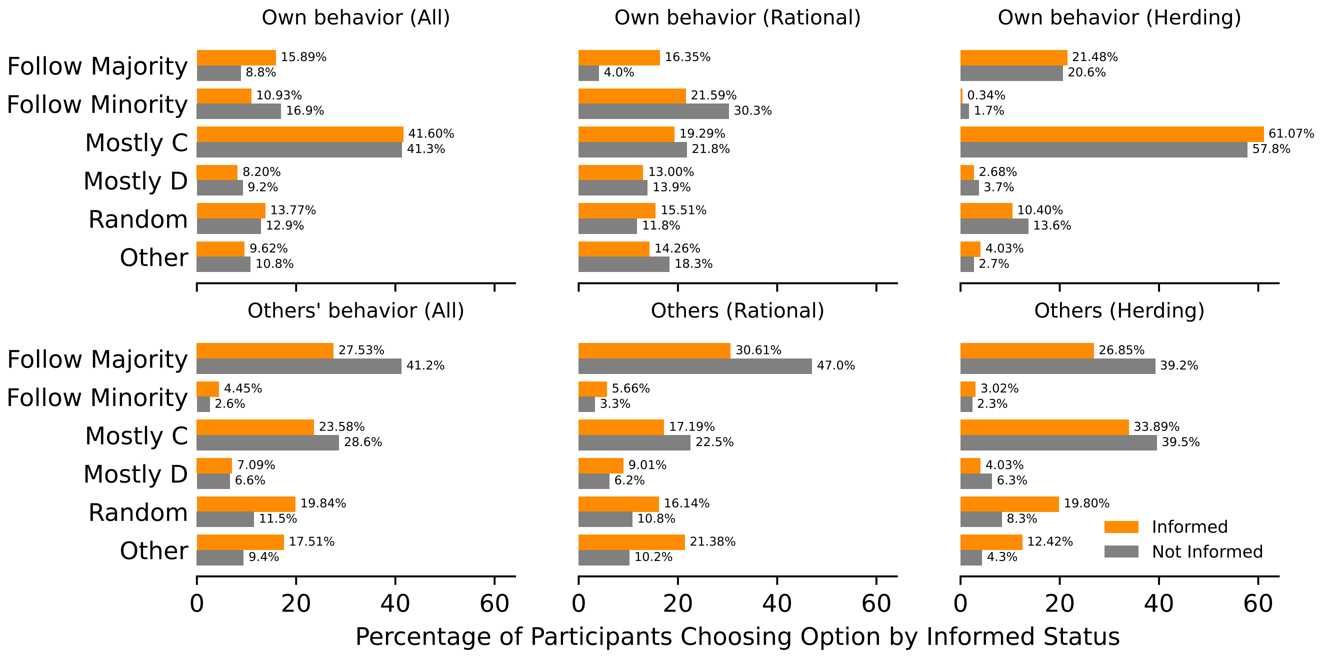

Fig. S3. Comparison of participants' beliefs about personal and others' behaviors under informed and not-informed conditions.

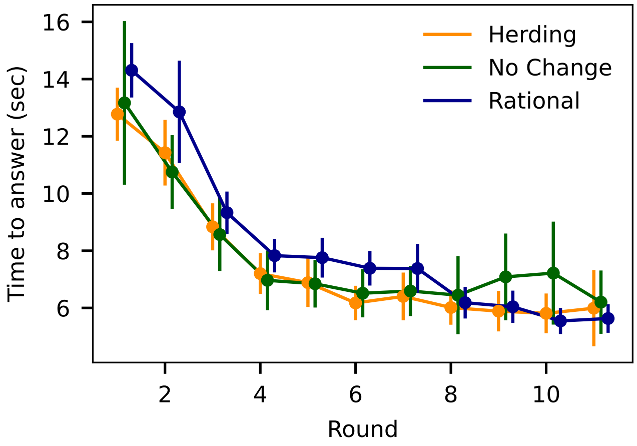

Fig. S4. Average decision-making time for each participant per round, accompanied by a 99% standard error around the mean. This figure illustrates how the time to answer varies across different rounds of the game, reflecting the complexity and participants' engagement with the task.

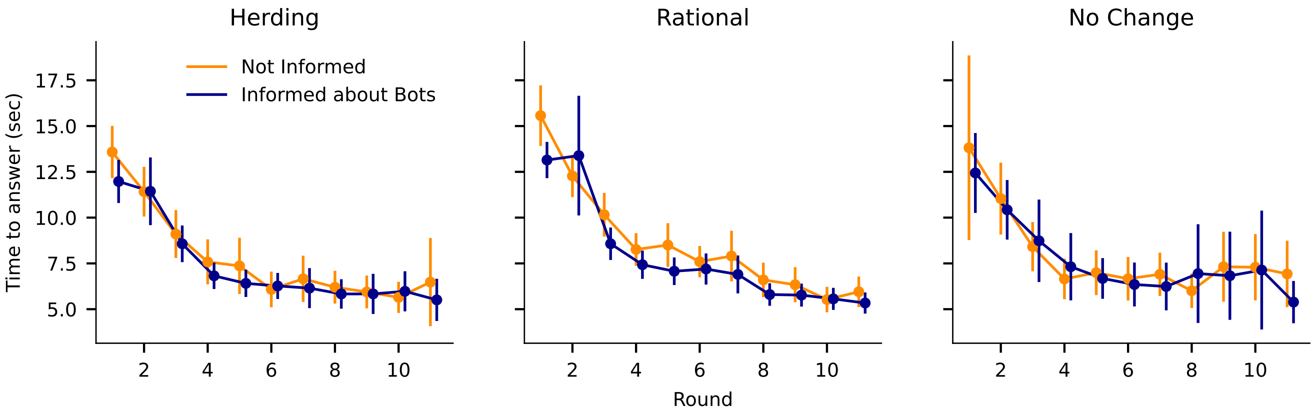

Fig. S5. Average decision-making time for each participant per round, accompanied by a 99% standard error around the mean. The orange line denotes response times for participants unaware they were engaging with automated players (bots), while the blue line denotes those who were informed of this fact.

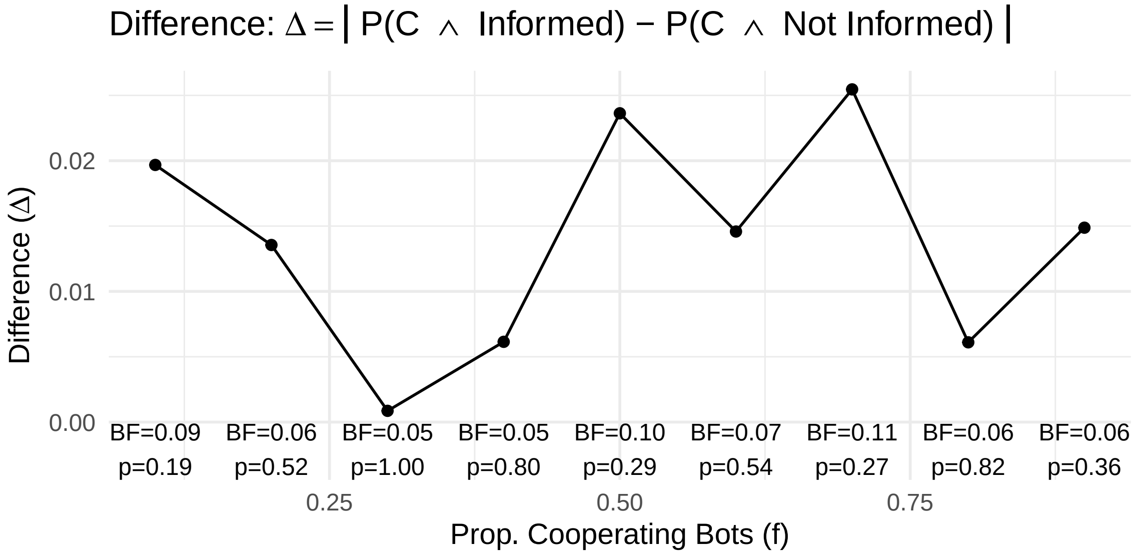

Fig. S6. Difference in the proportion of participants choosing to cooperate, denoted as $P(C)$, between the 'Informed' and 'Not Informed' conditions across varying levels of bot cooperation. Annotations below each point indicate the p-value from the chi-square test and the Bayes Factor (BF), which provide measures of statistical significance and evidence strength, respectively.

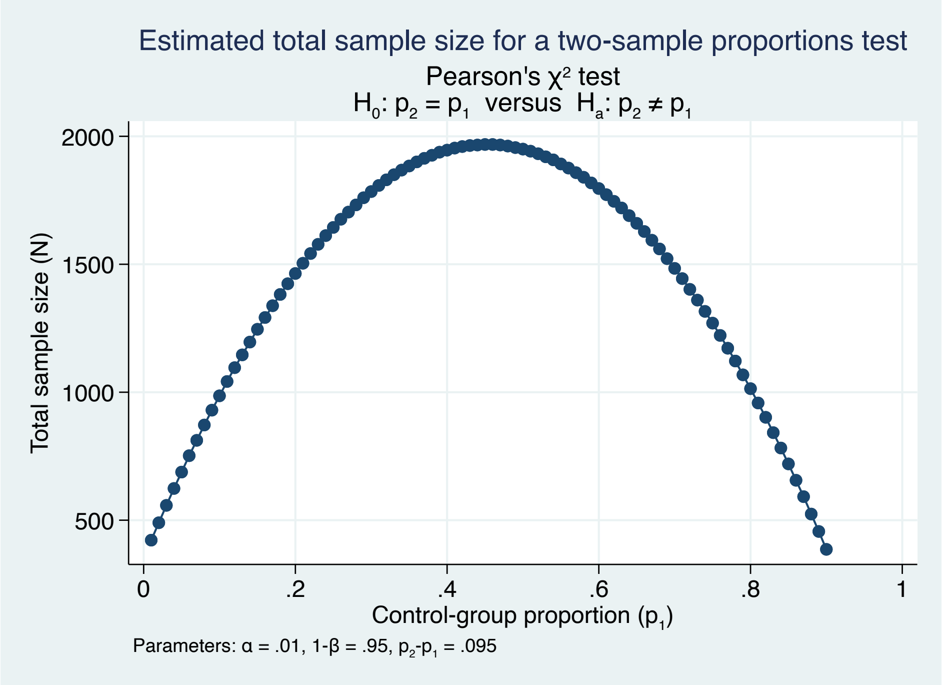

Fig. S7. Required Sample Size for Detecting a 9.5% Difference in Probability to Cooperate ($(P(C))$) using a χ^2 test with `twoproportions` in Stata. The chart illustrates that a minimum sample size of 2,000 is needed when the control group's $P(C)$ is 0.5. Assumptions include a statistical power of 0.95 (1-β) and a significance level of 0.01 ((α)). Note that the x-axis labeled "Control-group proportion" corresponds to our $P(C)$ for the informed group, i.e., the proportion of participants choosing C when informed.

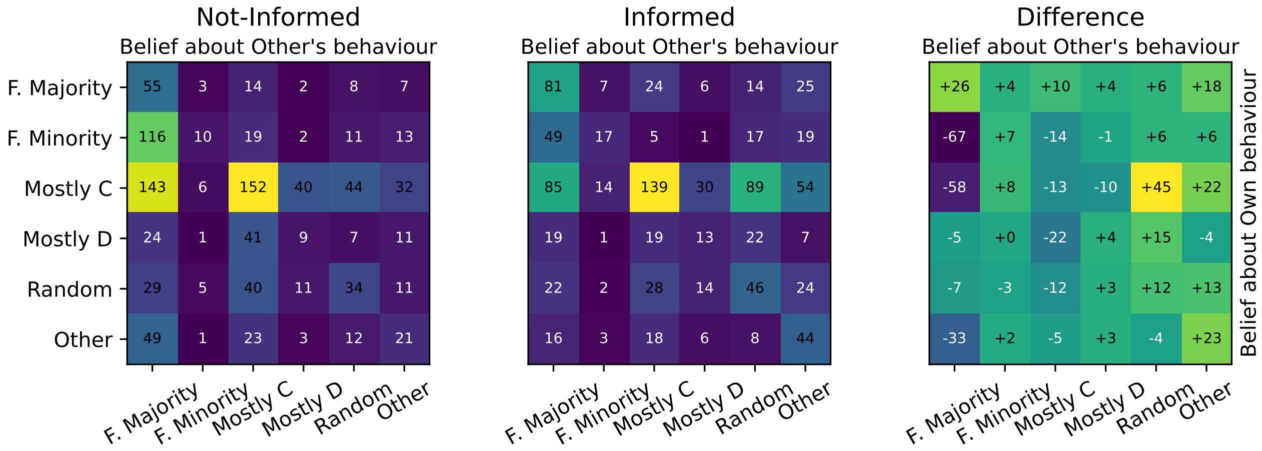

Fig. S8. Number of participants answering the debriefing question about their beliefs regarding their own behavior and that of others. Left panel: participants in the not-informed condition, middle panel: informed condition, right panel: difference in the number across conditions, # Difference = # Informed – # Not informed.
